# Do Consumers Change Their Perception of Liking, Expected Satiety, and Healthiness of a Product If They Know It Is a Ready-to Eat Meal?

**DOI:** 10.3390/foods9091257

**Published:** 2020-09-08

**Authors:** Laura Laguna, Beatriz Gómez, María D. Garrido, Susana Fiszman, Amparo Tarrega, María B. Linares

**Affiliations:** 1 Instituto de Agroquímica y Tecnología de los Alimentos (IATA, CSIC), Avenida Agustín Escardino Benlloch 7, 46980 Valencia, Spain; laura.laguna@iata.csic.es (L.L.); sfiszman@iata.csic.es (S.F.); 2 Facultad de Bromatología, Universidad de Entre Ríos, 2820 Gualeguaychú, Entre Ríos, Argentina; bgomez@fb.uner.edu.ar; 3 Departamento de Tecnología de Alimentos, Nutrición y Bromatología, Universidad de Murcia, 30003 Murcia, Spain; mgarrido@um.es (M.D.G.); blinares@um.es (M.B.L.)

**Keywords:** expected satiating capacity, healthiness perception, expected liking, ready-to-eat meal

## Abstract

A ready-to-eat meal is a prepared meal within a container or package that requires little preparation or heating before consumption. Despite ready-to-eat meals being widely consumed, to date, little information is available on the consumers’ perceptions of such products in comparison to a homemade meal. Thus, three groups of eighty participants took part in the present study; each group evaluated five ready-to-eat meals (*Pasta*, *Meatballs*, *Salad*, *Beans*, and a *Sandwich*) using one of the following conditions: (i) observation of the packaging, (ii) observation of the meal on a plate (photographs), and (iii) tasting the ready-to-eat product with the packaging being presented alongside the meal. Consumers were asked about their liking, satiety, and healthiness perception. The results showed that both the ready-to-eat pack and sensory quality of the product highly impact liking and healthiness perceptions. Being a ready-to-eat meal in a pack has a negative impact on liking expectations of the meal; however, the sensory quality can either counteract these effects or increase them. Expected satiety of meals depends on the type of meal and varies slightly according to the evaluation condition.

## 1. Introduction

“Ready meal” or “ready-to-eat”, as terms, can apply to a packaged full meal or main course comprising meat, fish, or vegetables and requiring little preparation and cooking [1]. Therefore, when consumers buy a ready meal, the ingredients have been already transformed and are presented in a pack where consumers can normally see the product, either by a colour printed image or through a transparent window on the packaging, i.e., *sandwiches* and *salads* [2].

According to the Spanish Association of Manufacturers of Prepared Dishes, in 2018, the consumption of ready-to-eat meals in Spain was 14.64 kg per person, representing an increase of 2.7% from the previous year [3]. Other countries such as the UK and USA had higher consumption; in 2018, their consumption was 19 and 27.8 kg per person, respectively [4].

The increase in popularity of ready-to-eat meals has been attributed to the scarcity of time in which to perform households chores [5]. A lack of time for cooking and the cost-effectiveness of such products have caused the ready-to-eat meals market to grow [6,7], and in Western societies, ready-to-eat meals have become, and continue to be, an essential part of consumers’ lives.

To further understand the importance of convenience for consumers when choosing ready-to-eat meals, some studies have focused on analysing the types of consumers that buy ready-to-eat meals according to different categories, i.e., demography, economy, education, and income [8,9]. However, nowadays the consumption of ready-to-eat meal is not only tied to households with two working parents. Further studies also showed the motivation that drives consumers to choose ready-to-eat meals; it was found that the choice between cooking or eating a ready-to-eat meal depended on the degree of convenience of the product, its sensory characteristics, and its perceived healthiness [10]. These characteristics are not unique ready-to-eat meals, but apply to all food products.

The Total Food Quality Model [11], a general framework for analysing food choice and quality perception, distinguishes between expected and experienced quality inferred from informational cues and actual experience, respectively. This model proposes four major dimensions that cover the principal aspects of food quality: taste (but also smell and appearance), health, convenience, and process. The first one is the hedonic quality dimension, and can usually only be determined after consumption. The second one is related to how consumers perceive a food product will affect their health that is mostly based upon consumer trust. Convenience is related to saving time and physical or mental energy in the process of planning, buying, storing and preparing the food, its consumption, cleaning, and disposing of the leftovers. Finally, process is related to the how consumers perceive a food has been produced (organic production, animal welfare, etc.).

Consumer attitudes and beliefs and/or previous experiences [12] contribute to their expectations. For a given ready meal, trust and the association with the homemade version help consumers create an idea about the product. Therefore, when finally consuming the meal, the actual product characteristics should also match the expected ones.

A particular feature of ready-to-eat meals is that in a supermarket, they are presented as convenient, i.e., precooked and packaged. Therefore, the packaging has an impact on quality perception. As previously reported, there are many packaging factors (images, shape, colour, and orientation of packaging elements) [13,14], as well as information such as the ingredients, nutrition facts, etc., that impact consumer quality expectations.

Regarding healthiness perception, nutritional studies have reported that ready-to-eat products contain more fat, sugar, and salt than homemade meals [15,16,17,18,19,20,21].

Food satiety perception is a dimension not included in the aforementioned model [11]. As ready meals are sold as individual, full-portion-sized packs, perception of satiety can be especially relevant. Part of consumers’ opinions can be based upon fulfilling immediate and future needs to bridge the time until a next meal. It has been shown that consumers’ expected satiety judgements are based on the energy content of food, but that they are also related to a learned value based on previous experience [22], as well as being influenced by other factors, such as familiarity and sensory properties [23].

Both quality expectation and experience (before and after consumption, respectively) are believed to determine the degree of satisfaction with a product by a confirmation or disconfirmation process [24]. So, comparisons between before and after consumption experiences will contribute to understanding product quality perception and consumer satisfaction. At present, little information is available on the role of packaging and the sensory properties of ready-to-eat meal products regarding (expected) liking, satiety, and healthiness perception compared to what consumers would expect from a homemade meal.

The initial hypotheses of the present study are that knowing that a food item is a packaged ready to eat meal negatively affects consumer perception, and that the sensory characteristics of the product can modulate this perception. Comparing the perception of ready to eat products in different conditions can broaden our understanding of consumer responses.

Therefore, consumers’ (expected) liking, expected satiety, and healthiness perception of five ready-to-eat meals under different conditions (visually as a meal on a plate, visually as a meal pack, and tasting the meal) were evaluated to determine the impact of the “ready-to-eat” concept and the product’s sensory characteristics on consumer response.

## 2. Materials and Methods

### 2.1. Ready-to-Eat Meals

Five different ready-to-eat meals were bought in a local supermarket: *Pasta* (carbonara macaroni), *Meatballs* (garden meatballs), *Salad* (New Yorker salad), *Beans* (*Fabada*, a traditional Spanish bean stew), and *Sandwich* (lettuce and egg). Table 1 shows the ingredients and describes the packaging material of the different ready-to-eat meals. The ingredients, weight, and nutritional facts as they are displayed on the packs can be found in Table A1 (Appendix A).

### 2.2. Consumer Tests

Individuals over 18 years of age were invited to take part in the study. Three separate groups of eighty participants with similar characteristics concerning gender (64, 66, and 68% women), age (36, 37, and 35 years on average), and age distribution (60% between 18–40 years of age and 40% older than 40) took part in a sample evaluation. People following any restrictive diet and smokers were excluded. No other demographic features were taken into account.

**Figure 1:**
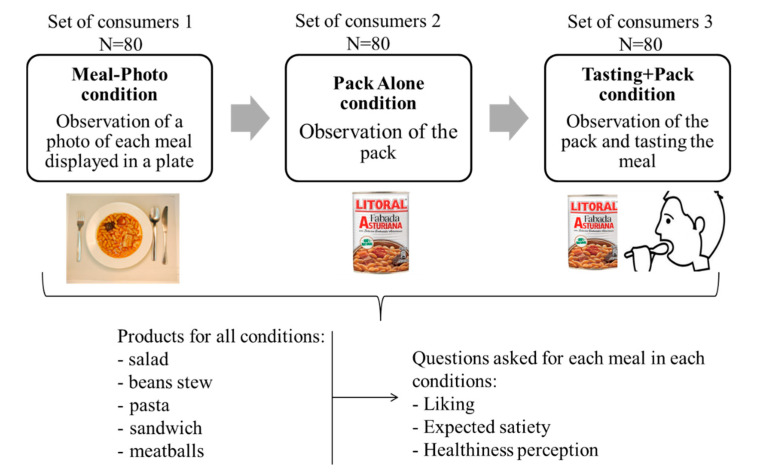
Scheme of the experimental procedure where three different sets of consumers participated in one of the three different conditions and evaluated five meals.

Figure 1 shows a scheme of the experimental procedure: each group of 80 participants evaluated the five meals under one of the following conditions:

(i) Meal-Photo condition: The participants were asked to observe a photograph of the meal (i.e., the complete contents of the pack) put on a white plate with a knife and fork on the sides. In this setting, the consumers were not aware that the meals were ready-to-eat products. The photographs were taken immediately after cooking/serving the dish (Table 2) using a Nikon D800 (36 Mpx) camera (Nikon GmbH, Düsseldorf, Germany) with a Nikon 50 mm f/1.8 lens(Nikon GmbH, Düsseldorf, Germany) mounted on a Manfrotto tripod (Nikon GmbH, Düsseldorf, Germany) perpendicular to the plate and at the same distance from it. A Nikon SB900 flash (Nikon GmbH, Düsseldorf, Germany) mounted in a soft white umbrella (diameter: 80 cm) at an angle of 45° was also used. The Exchangeable image file format (Efix) data were as follows: focal length = 50 mm; exposition = 1/60 s; aperture = f/8; ISO = 100; colour temperature = 5800 K; flash power = 1/32. Basic adjustments to the RAW files (levels, curves, colour saturation, lens correction, and colour temperature) were made with the Lightroom 4.4 software (Adobe Systems Inc., San Jose, California, USA)). The photographs were coded with three digits using the Photoshop CS6 software (Adobe Systems Inc., San Jose, California, USA). Finally, seven groups of eight photographs were printed (240 pp resolution) on matt photographic paper (Granate Laboratorio, Molina del Segura, Spain) measuring 31 cm × 40 cm to maintain the original size of the dishes, and were mounted on foam sheets (thickness: 5 mm).

(ii) Pack Alone condition. The participants were asked to observe the unopened pack of the products. They were free to touch and to inspect the pack. In this condition, they were aware that the meal was ready-to-eat.

(iii) Tasting+Pack condition. The participants were asked to taste the product with an identical, unopened pack of the same product in view; in this condition, the participants knew that the meal was a ready-to-eat product, and were free to touch and inspect the pack. The participants were presented with a small plate and meal portion and were asked to taste it. Five different dishes were prepared appropriately, according to the type of dish (heated for the *Pasta*, *Meatballs*, and *Beans* or served at room temperature for the *Sandwich* and *Salad*) and put on a plate with a knife and fork on the sides. Approximately 80–100 g was served, with all the plates having approximately the same number of calories.

Under each condition, the five samples (photograph of the meal, pack, and product tasted) were presented to the consumers in randomised order.

Consumers were asked to rate their (expected) liking, expected satiety, and healthiness perception for each sample. Liking was evaluated liking using a 9-point hedonic scale ranging from 1 (“dislike very much”) to 9 (“like very much”). Expected satiety was evaluated with a 7-point scale [25] ranging from 1 (“this meal would not satiate me, I will be hungry straight away”) to 7 (“this meal would be very satiating, it will allow me to fast until dinnertime or more”). Healthiness perception was evaluated on a 9-point scale from 1 (“not very healthy”) to 9 (“very healthy”).

All subjects gave their informed consent for inclusion before they participated in the study. The study was conducted in accordance with the Declaration of Helsinki, and the protocol was approved by the Research Ethics Committee of the Universidad de Murcia (Project ID 2933/2020).

### 2.3. Data Analysis

For each evaluation condition, the individual consumers’ liking scores of the five products were analysed by the internal preference mapping method, obtained by applying a PCA to the liking values of each of the five products with consumers as a variable. The results were expressed as scatter plots of samples and individual consumers in relation to the first two dimensions.

Also, for each evaluation condition, the effect of product on liking, expected satiety, and healthiness perception were studied by two-way ANOVA. A Tukey test was used for post hoc mean comparisons.

For each pair of conditions, the differences between the mean scores of (expected) liking, healthiness perception and expected satiety were studied using Student’s *t*-tests (*p* ≤ 0.05).

Data analyses were conducted using the statistical software package XLSTAT (Addinsoft, Barcelona, Spain, version 2018.2).

## 3. Results and Discussion

### 3.1. Consumer Liking

**Figure 2:**
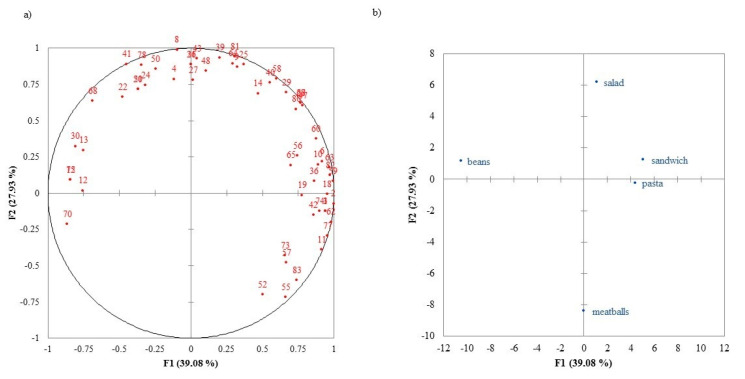
Internal preference map of consumer liking scores of the Meal-Photo condition: (**a**) consumers; (**b**) samples.

**Figure 3:**
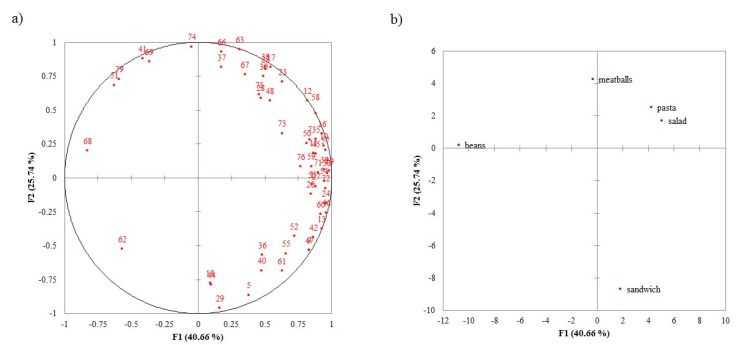
Internal preference map of consumer liking scores in the Pack Alone condition: (**a**) consumers, (**b**) samples.

**Figure 4:**
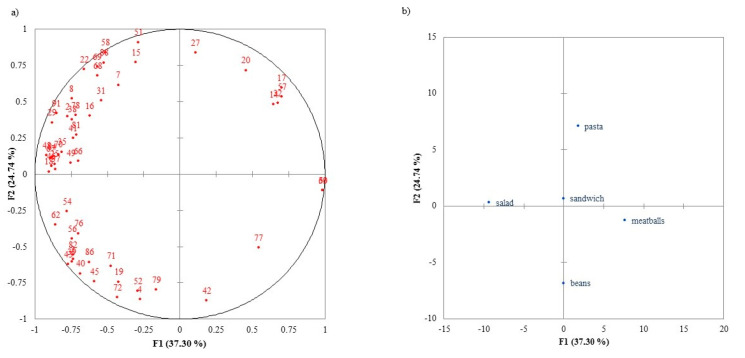
Internal preference map of consumer actual liking scores in Tasting+Pack condition: (**a**) consumers, (**b**) samples.

Internal preference maps in the three conditions (Meal-Photo, Pack Alone, and Tasting+Pack) are displayed in Figure 2, Figure 3 and Figure 4, respectively. The preference map corresponding to the evaluation of the photograph of meal on a plate (Meal-Photo condition) (Figure 2) explains 67.02% of the consumer variability. The first dimension (F1) separated *Pasta* and *Sandwich* from *Beans*, while the second (F2) separated *Salad*, on the top of the map, from *Meatballs*. Most consumers appeared on the top, right-hand side of the map, showing their preference for *Pasta*, *Sandwich* and *Salad* and rejection of *Beans* on the left. Consumers that preferred *Beans* or *Meatballs* were few.

The internal preference map for consumers’ expected liking when evaluating the product as a ready-to-eat meal (Pack Alone condition) is shown in Figure 3. The first two dimensions explain 66.40% of the variability. Most consumers, placed on the right side of the map, showed higher liking expectation for the *Salad* and *Pasta*, and lower liking expectation for *Beans* on the left half of the map. Few consumers had high expectations for *Meatballs* (on the top of the map); likewise, few had high expectations for the *Sandwich* (on the bottom of the map). Both groups were separated by F2.

Figure 4 shows the distribution of products according to the actual consumer liking after tasting the ready-to-eat meals, with the corresponding packaging in view (Tasting+Pack condition). The preference map explains 62.04% of the variability. F1 shows the preference of the consumers for *Salad*. F2 separates the traditional heartier foods (*Meatballs* and *Beans*) from the *Pasta*, *Salad*, and *Sandwich*. *Salad* was the preferred choice of most consumers, but the group was divided according to dislike for the other meals. The group on the top left quadrant disliked food that is hearty, while the group of the bottom left quarter disliked the *Pasta*.

These results show that the preference patterns were similar among consumers (i.e., they are not scattered all over the map) under the same condition; however, these patterns differed between conditions.

### 3.2. Effect of Evaluation Condition on the Liking Scores

The two-way ANOVA results showed that the two effects studied (sample and condition) were significant for the liking scores (*F* = 25.51, *p* < 0.001 and *F* = 17.81, *p* < 0.001, respectively). The interaction was also significant (*F* = 3.61; *p* < 0.001), indicating that the differences between the samples depended on the condition, and differences between conditions depended on the samples.

In the evaluation of the products as a photograph of the meal on a plate (Meal-Photo condition), participants were not aware that the meal was a ready-to-eat product; they evaluated the expected liking for the meal itself. Values ranged from 5.8 to 7.5 (Table 3); *Salad* and *Pasta* obtained the highest expected liking mean values, and *Beans* received the least expected liking values.

When evaluating the meal as a ready-to-eat product (Pack Alone condition), expected liking values were lower (4.7 to 6.8). *Salad* elicited the highest expected liking values, while *Beans* received the lowest. The Spanish word used in liking evaluations (“gustar”) has a heavy hedonic connotation. However, a more specific, food-related word like “tastiness” (“sabroso” in Spanish) could have been used, taking into account some previous findings [26] that showed that tastiness affects human response differently than preference (and healthiness). 

After tasting the meals with the packaging in view, liking values varied from 5.3 to 7.4. *Salad* was most liked; it was significantly higher than the rest of products, which did not significantly differ.

**Figure 5:**
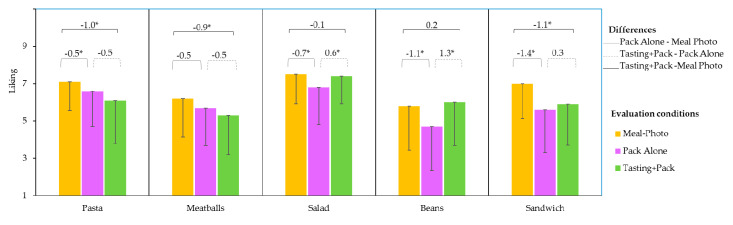
Liking mean values of each of the five meals evaluated on a plate (Meal-Photo condition), in the pack (Pack Alone condition), and after tasting with the packaging in view (Tasting+Pack condition). Differences between conditions (Pack- Meal Photo, Tasting Pack-Pack and Tasting pack- Meal Photo) are indicated with line segments over the top of the corresponding columns; significant differences are marked with an asterisk (*). Only the negative part of the standard deviation bars are shown for clarity.

To evaluate how the concept of ready-to-eat meals affected the liking expectation, the score subtractions of the mean values in different conditions and their significance were analysed (Figure 5). Comparisons between Meal-Photo and Pack Alone conditions indicated whether knowing that a meal was a ready-to-eat product affected liking expectations. All products showed a significant decrease in expected liking values (except *Meatballs*, whose decrease was not significant) when they were evaluated in their packaging. These results indicate that consumers had a bad perception of packaged ready-to-eat products from the meal photographs, or they expect the industrial product to be worse than a homemade or restaurant meal. Previous studies also found that in comparison with homemade meals, chilled ready-to-eat meals were less desirable and associated with lower sensory quality [27]. Beyond expected liking, other factors could explain the decrease in the liking scoring. One might be the awareness of the presence of additives, which appeared in the ingredients lists. The ready-to-eat meals contained food additives (Table 1) that are generally linked with unnatural or artificial and unhealthy substances [28,29,30]. Furthermore, in this study, consumers could associate the presentation (Meal on a plate photo) with a homemade meal. Moreover, a previous study showed that when consumers are involved in the preparation of a meal, there is an increase in liking scores [31]; therefore, it is likely that if the presented ready-to-eat meals had required more consumer involvement, they would have received higher liking scores. Although the ready-to-eat meals were presented with the pack displaying the list of ingredients, they have undergone unknown industrial processes, increasing consumer distrust [32].

The extent of the decreased liking was different depending on the meal, and was apparently related to the type of packaging. The decrease in expected liking was low for *Meatballs* and *Pasta*, which were presented in a plastic tray that is designed to simulate a plate, with a cardboard envelope that could project the idea of a better-quality product.

The highest decrease in expected liking mean values corresponded to the *Sandwich* and *Beans*. Packaged sandwiches in a triangle pack (well-known in European countries) are an incipient meal modality in Spain with a narrow range of variety in terms of brands, fillings, and type of bread. The poor result can therefore be attributed to a lack of confidence in this industrial product. In Spain, sandwiches are a familiar meal concept (“bocadillo”) which is available and freshly prepared in cafeteria-like restaurants or at home; however, consumers are not familiar with sandwiches as a ready-to-eat, packed product, which could explain the impact on liking. In contrast, *Beans* are a traditional, usually homemade Spanish meal (“Fabada”). The fact that they were packaged in a can probably led to the association with food of lesser quality compared to the other types of packaging. Previous studies have shown that consumers usually perceive canned food as being more processed than other ready-to-eat products [33,34].

To evaluate the impact of tasting on the liking of the ready meal products, the differences between the scores obtained when tasting the product with the Tasting+Pack condition and those with the Pack Alone condition were analysed (Figure 5). In addition, the differences between the scores obtained in the Tasting+Pack and Meal-Photo conditions were also studied to determine whether the ready to eat product (Tasting+Pack condition) met the expectations raised by the meal on a plate (Figure 5). 

When tasted, the *Beans* and *Salad* mean scores increased significantly compared to the expectation created by the corresponding pack (Figure 5). For these products, which saw a significant drop in the expected liking values when presented in the pack, liking values increased after tasting, coming in line with what consumers expected from the meal on a plate photo. These results could be attributed to the sensory quality of products that can counteract the “ready meal” effect; in other words, the product (Tasting+Pack) met the hedonic expectations of consumers. *Beans* presented the highest recovery effect (actual liking was even slightly higher than expectation after having seen the meal on a plate). 

For *Sandwich*, tasting increased the liking value slightly with respect to the pack evaluation, but the difference was not significant. Sensory quality was found to be insufficient to increase liking, which remained significantly lower than what consumers had expected when observing the meal on a plate. For *Pasta* and *Meatballs*, tasting slightly decreased liking with respect to pack evaluation, but the difference was not significant. However, liking values for these products when tasted were significantly lower than expected from the meal due to the conjoint effect of the packaging and poor sensory quality.

Thus, both the Pack Alone and Tasting+Pack conditions influenced liking. Packaging has, in general, a negative impact on meal liking expectations, but sensory quality can influence this in either a positive or a negative way. Therefore, both effects need to be considered to fully understand consumer liking for ready-to-eat products.

### 3.3. Effect of Evaluation Conditions on Expected Satiety

The two-way ANOVA results showed that the sample and evaluation conditions had significant effect (*F* = 174, *p* < 0.0001 and *F* = 3.4, *p* = 0.034, respectively) on expected satiety values; the interaction between both effects was also significant (*F* = 2.1, *p* = 0.028). However, most of the variability depended on the sample, while the effects of condition and their interaction were less significant.

Expected satiety scores were significantly different between samples when a photograph of the product on a plate was evaluated (Table 4). Consumers considered the *Sandwich* to be the least satiating, with a mean score of 3.5, implying that they would expect to be hungry and need to eat again by mid-afternoon.

For the *Salad*, expected satiety showed an intermediate mean value (4.0, enough to last until mid-afternoon), while the *Pasta*, *Meatballs* and *Beans* were considered more satiating, with mean scores between 5.3 and 6 (enough to last until dinner).

The patterns of differences in expected satiety between products were similar for the other two conditions, i.e., where meals were evaluated having the pack present (Pack Alone and Tasting+Pack conditions), in which the *Sandwich* and *Salad* obtained the lowest mean values (Table 4). Previous authors have shown that one of the most fundamental factors in satiety is the energy content of the meal [22,35]. However, on the packaging, consumers had information concerning the ingredients, caloric content, and nutritional composition; still, the belief that *sandwiches* and *salads* were “light” meals influenced the expected satiating scores, even though the energy (Kcal) values were similar between the five dishes. This showed that consumers tend not to pay attention to the information on the label, believing that *salads* and *sandwiches* are less caloric. A previous study [36] showed how a pasta salad labelled as “healthy” or “hearty” influenced self-reported satiety, consumption volume, and subsequent consumption of another food. *Pasta*, *Meatballs* and *Beans* are typically served warm and were probably seen as being more complete, satiating meals.

**Figure 6:**
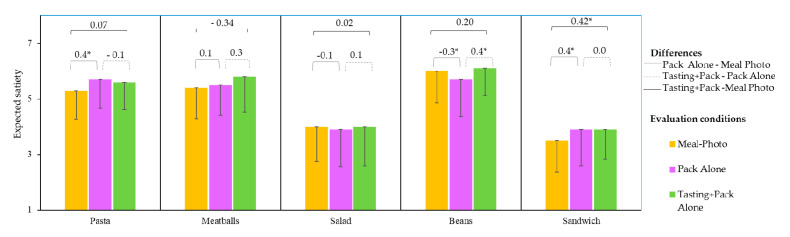
Expected satiety mean values of each meal evaluated on a plate (Meal-Photo condition), in the pack (Pack Alone condition), and after tasting with the packaging in view (Tasting+Pack condition). Differences between conditions (Pack- Meal Photo, Tasting Pack-Pack and Tasting pack- Meal Photo) are indicated with line segments over the top of the corresponding columns; significant differences are marked with an asterisk (*). Only the negative standard deviation bars are shown for clarity.

Regarding the differences between the evaluation conditions, as shown in Figure 6, the *Meatballs* and *Salad* did not show significant differences in satiety perception under any of the conditions. For *Pasta* and *Sandwich,* expected satiety was higher in the Pack Alone condition than in the Meal-Photo condition. A difference for *Sandwich* was also observed when comparing the Tasting Pack and the Meal-Photo conditions. For *Bean,* the expected satiety was lower when evaluating the packaging alone, but this increased significantly when the product was tasted (Tasting+Pack condition), achieving a value that was similar to that expected from the meal on the plate photo. The *Beans* were canned, and the contents were perceived as being smaller on the pack than on the plate; however, the sensory characteristics of *Beans*, related to the rich, strong flavours in this meal, may have reinforced or evoked satiation in consumers, allowing them to achieve a similar score to the expected one based upon looking at a photograph of the meal on a plate. Both flavour and thickness have previously been shown to enhance satiety expectations [37].

These results indicate that expected satiety scores were associated with the actual presented meal, rather than whether it was a commercial ready-to-eat product or not, or based upon its sensory properties. Previous authors have shown that consumers have ideas about satiation for each kind of meal based on previous eating experiences [38,39].

Although previous studies [22] have suggested links between consumer scores of expected satiety and liking (among other factors), in the present study, for all conditions, no significant relationship was found between these variables.

### 3.4. Effect of Evaluation Conditions on Healthiness Perception

Table 5 shows the scores of healthiness perception of the five products under the three conditions. The ANOVA results indicate that healthiness perception varied depending on the product, evaluation condition, and their interactions (*F* = 19.85, *p* < 0.0001; *F* = 75.31, *p* < 0.0001; *F* = 2.44, *p* = 0.013, respectively).

In the three conditions, *Salad* was considered the healthiest meal, and *Pasta and Meatballs* the least healthy.

**Figure 7:**
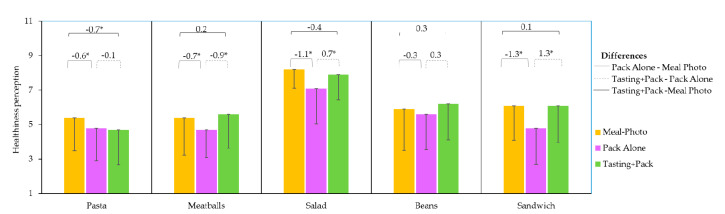
Healthiness perception mean values of each of the five meals evaluated on a plate (Meal-Photo condition), in the pack (Pack Alone condition), and after tasting with the packaging in view (Tasting+Pack condition). Differences between conditions (Pack- Meal Photo, Tasting Pack-Pack and Tasting pack- Meal Photo) are indicated with line segments over the top of the corresponding columns; significant differences are marked with an asterisk (*). Only the negative part of the standard deviation bars are shown for clarity.

The perception of healthiness values were significantly lower in the Pack Alone conditions than those obtained through looking at the meal on a plate (Meal-Photo condition), except for *Beans*, which did not significantly decrease (Figure 7). These results confirm that in consumers’ minds, processed or prepared meals are less healthy than homemade versions. The extent of the difference between the perception of healthiness under these two conditions depended on the product; the highest drop seen (1.3) was for the *Sandwich*, which was considered healthier on the plate than in the Pack Alone. *Salad* also showed a strong decrease in healthiness perception (1.1) when evaluated in the Pack Alone condition, when the participants were aware of its being a ready-to-eat product. However, the *Salad* was still considered the healthiest product (7.1).

One or more elements of the packaging can also contribute to such differences (this was not the focus this study). Still, since all products underwent a decrease in healthiness perception when observed in their packaging, changes in perception seem to be based on the preconceptions that consumers had towards commercial, ready-to-eat meals, as opposed to their energy content or nutritional value. Previously, studies have shown how ready-to-eat meals have been associated with high levels of fat, sugar, and salt [15,16,17,18,19,20,21]. However, nowadays it cannot be assumed that all homemade meals are healthier than their ready meal counterparts [40].

Table A1 shows that *Salad* was the meal with the highest fat and saturated fat content (because of the sauce, bacon, and cheese); however, it scored as the healthiest, because of the belief that a *Salad* is a light meal. In addition, a previous study reported that consumers have more difficulty perceiving the healthiness of a whole meal than they do when inspecting the individual ingredients [41].

Further study on using different elements in the packaging design could counterbalance the negative perceptions that consumers have about ready meals. Also, study of the variations between products of the same category with fewer and more controlled differences (e.g., brand, kind of package, and subtler differences in ingredients) should be conducted.

Tasting also had an impact on healthiness perception, even with the knowledge that the meals were ready-to-eat products (Tasting+Pack condition). After tasting, the healthiness perception of *Sandwich*, *Meatballs*, and *Salad* significantly increased compared to the Pack Alone condition, while the values did not significantly differ from those in the Meal-Photo condition. This effect seems to be linked a product’s sensory qualities, which can disconfirm the initial, low expectations that can result from seeing the packaging. Comparing the meal on a plate (Meal-Photo condition) and the tasted meal (Tasting+Pack), only *Pasta* showed a decrease in the healthiness score.

## 4. Conclusions

This study aimed to determine whether consumers change their (expected) liking, expected satiety, and healthiness perception when they learn that a meal is ready-to-eat. The evaluation of the meal on a plate, with no indication that it was a commercial, ready-to-eat meal, was included as a way of evaluating consumer expectations regarding the meal, which were then compared to ready-to-eat meal products. These comparisons allowed us to gain a fuller understanding of consumers’ responses to this category of products. Knowing the product was a commercial, ready-to-eat one had a negative impact on liking expectations and healthiness perception compared to when the meal was presented on a plate and consumers were not aware that they were seeing a commercial, ready-to-eat product. This could be explained by the fact that homemade meals are still considered more likeable and healthier, as they do not contain additives that consumers consider a health risk, and are not industrially processed. Additionally, if ready meals required more consumer involvement, they would receive a higher liking score, and consumers would be more trustful. Furthermore, if consumers could read and understand nutritional information concerning homemade meals, their healthiness perception of ready meals might improve.

The sensory quality of ready-to-eat meals can counteract the negative impact on liking and healthiness perception. Therefore, a ready-to-eat product with optimised sensory qualities can be a liked and perceived healthy product, such as might be expected from a homemade or restaurant-bought meal. Satiety expectations were defined by the type of meal and were slightly affected by the packaging information and sensory properties. Future studies are needed to determine the elements (characteristics on the packaging and sensory properties) that maximise liking, healthiness, and satiety perception to optimise both the sensory and packaging properties of ready-to-eat products.

As a limitation, in the present study, consumer variables other than age and gender were not controlled. Some other demographic characteristics might have influenced consumer responses.

## Figures and Tables

**Table 1:** List of ingredients and packaging description of the ready-to-eat meals.

Meal	List of Ingredients	Packaging Material	Images on the Pack
Pasta	Pasta, carbonara sauce *, onion, smoked bacon **	White plastic tray cover with opaque plastic film inside cardboard envelope	Pasta served on a dish
* Carbonara sauce ingredients: Water, cream powder (dairy), sunflower oil, salt, modified corn starch, flavour (dairy), smoke flavour, stabiliser (xanthan gum), natural flavour, white pepper, and soy lecithin ** Smoked bacon ingredients: Bacon pork, water, salt, sugar, stabiliser (e-451), antioxidant (e-316), aroma of natural smoke, and spices			
Meatballs	Pork Meatballs *, tomato sauce **, vegetables [potato, peas, carrot, onion, mushroom]	White plastic tray cover with opaque plastic film inside cardboard envelope	Meatballs served on a dish
* Meatball ingredients: Lean pork, water, white breadcrumbs (wheat flour (gluten), water, salt, and yeast), egg, salt, onion, garlic, and parsley ** Tomato sauce ingredients: Tomato, water, wine, sugar, modified corn starch, olive oil, natural flavour (gluten and soy), salt and, black pepper			
Salad	Curly escarole, radicchio, canon, Modena and honey sauce *, bacon **, onion ***, gouda, cheddar cheeses	Transparent plastic tray	No images presented, but the salad ingredients can be seen through the packaging
* Modena and honey sauce: Water, sugar, Modena balsamic vinegar (5%), honey (3%), colouring (E150c), salt, sunflower oil, acidifier (E260), stabiliser (E415), preservative (E202) ** Bacon: Pork belly, salt, sugar, corn dextrose, flavourings, stabilisers (E451, E407), flavour enhancer (E621), antioxidant (E316, E331), preservative (E250), coating agent (edible gelatin, E200), frying oil (sunflower), and antioxidants (E320, E321) *** Onion (7%): Onion, vegetable oil (palm), wheat flour, salt. Toasted wheat bread cubes: wheat flour (86%), vegetable oil (sunflower), dextrose, wheat gluten, yeast, salt, buttermilk, barley malt extract, and antioxidant (ascorbic acid)			
Beans	Beans, water, chorizo *, black pudding **, smoked bacon ***, pig lard, salt	Tin can	Beans served on a traditional casserole-like dish
* Chorizo: Lean meat, chin and smoked pork belly, salt, paprika, and garlic ** Black pudding: Pig lard, pig chin and smoked bacon, blood and lean pork meat, onion, paprika, salt, and oregano *** Smoked bacon: Pork belly, salt, and natural smoke			
Sandwich	Bread *, tomato, lettuce, mayonnaise, hard-boiled egg, soybean oil, water, EGG yolk, vinegar, sugar, salt, starch, flavour, concentrated lemon juice, antioxidant E-385, colourants (paprika extract, E-160a)	Transparent plastic film and black plastic triangle	No images presented, but the sandwich and filling can be seen through the package
* Bread ingredients: wheat flour, water, yeast, sunflower oil, sugar, salt, WHEAT gluten, preservatives (E-282, E-200, E-202), starch, and soya flour			

**Table 2:** Photographs of the ready-to-eat meals presented to the participants (Meal-photo condition).

**Meatballs**	**Salad**		**Beans**
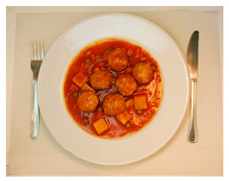	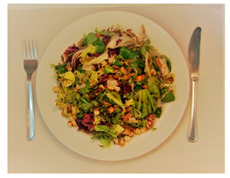		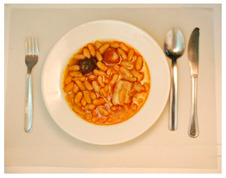
**Pasta**		**Sandwich**	
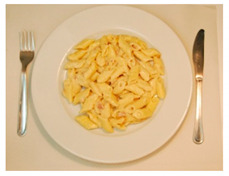		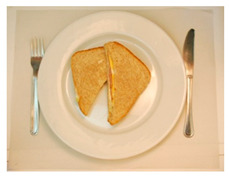	

**Table 3:** Liking mean values, and standard deviation in parenthesis, of the five products evaluated on a plate (Meal-Photo condition), in the pack (Pack Alone condition) and after tasting with the pack in view (Tasting-Pack alone condition).

Product	Scenario		
	Meal-Photo *	Pack *	Tasting-Pack *
Pasta	7.1 (1.5) ^a^	6.6 (1.9) ^a,b^	6.1 (2.3) ^b^
Meatballs	6.2 (2.1) ^b,c^	5.7 (2.0) ^b,c^	5.3 (2.1) ^b^
Salad	7.5 (1.6) ^a^	6.8 (2.0) ^a^	7.4 (1.5) ^a^
Beans	5.8 (2.3) ^c^	4.7 (2.3) ^d^	6.0 (2.3) ^b^
Sandwich	7.0 (1.8) ^a,b^	5.6 (2.3) ^c,d^	5.9 (2.2) ^b^

* Different superscript letters within the same column (condition) denote significant differences according to the Tukey’s test.

**Table 4:** Expected satiety mean values, and standard deviation in parenthesis, of the five products evaluated on a plate (Meal-Photo condition), in the pack (Pack Alone condition) and after tasting with the packaging in view (Tasting-Pack alone condition).

Product	Condition		
	Meal-Photo *	Pack *	Tasting + Pack *
Pasta	5.3 (1.0) ^a^	5.7 (1.0) ^a^	5.6 (0.9) ^a,b^
Meatball	5.4 (1.1) ^a^	5.5 (1.1) ^a^	5.8 (1.3) ^b^
Salad	4.0 (1.2) ^b^	3.9 (1.3) ^b^	4.0 (1.4) ^c^
Beans	6.0 (1.1) ^a^	5.7 (1.3) ^a^	6.1 (0.9) ^a^
Sandwich	3.5 (1.1) ^c^	3.9 (1.3) ^b^	3.9 (1.0) ^c^

* Different superscript letters within the same column (condition) denote significant difference according to Tukey’s test.

**Table 5:** Healthiness perception mean values, and the standard deviation in parentheses, of the five products evaluated on a plate (Meal-photo condition), in the pack (Pack alone condition) and after tasting with the packaging in view (Tasting-Pack alone condition).

Product	Condition		
	Meal-photo *	Pack *	Tasting Pack *
Pasta	5.4 (1.9) ^c^	4.8 (1.9) ^b,c^	4.7 (2.0) ^c^
Meatball	5.4 (2.2) ^c^	4.7 (1.6) ^c^	5.6 (2.0) ^b^
Salad	8.2 (1.1) ^a^	7.1 (2.0) ^a^	7.9 (1.5) ^a^
Beans	5.9 (2.4) ^b^	5.6 (2.0) ^b^	6.2 (2.1) ^b^
Sandwich	6.1 (2.0) ^b^	4.8 (2.1) ^b,c^	6.1 (2.1) ^b^

* Different superscript letters within the same column (condition) denote significant difference according to Tukey’s test.

## Appendix A

**Table A1:** Nutritional facts of the ready-to-eat products.

Product	Nutrition Facts (per 100 g of product)							
	Energy (kcal)	Fat (g)	Saturated Fat (g)	Carbohydrates (g)	Free Sugars (g)	Fibre (g)	Protein (g)	Salt (g)
Pasta	129	7.2	3	11.9	1.1	0.6	4.3	1.3
Meatballs	118	5.5	1.7	6.5	3.1	1	9.9	1.7
Salad	181	10.9	5.5	12.6	4.05	1.8	6.8	0.9
Beans	142	8.3	3.1	7.9	0.7	5.2	6.4	0.9
Sandwich	186	10	2	20	3.3	-	4.6	0.8
